# The Emmanuel Idea

**Published:** 1908-12-26

**Authors:** 


### The Hospital

A Journal of

The Medical Sciences and Hospital Administration.

New Series. No. 95, Vol. IV. [No. 1167, Vol. XLV.] SATURDAY, DECEMBER 26, 1908.

### The Emmanuel Idea.

A good deal has been printed in the daily Press recently about the new variant upon "Christian Science," which is being advocated in America by several clergymen in association, it is understood, with certain physicians. It is said, no doubt with accuracy, that the establishment of clinics is contemplated in connection each with some particular place of worship in different towns in the United States; and that these are to be modelled on that which has been formed by persons attached to the Emmanuel (or, as sometimes spelt, Emmanual) Church of Boston. Exactly and precisely how far the "Emmanuel Idea" may be a legitmate adjunct to the ordinary means of dealing with those psychic manifestations which are included in the somewhat catholic catalogue of functional disturbances is rather a large question, and before anyone can formulate opinions on many of the points which arise he must have fairly full information what this medico-clerical alliance claims, and how it sets to work. We say a medico-clerical alliance, because a good deal has been made of the adhesion of one or two neurologists to the doctrines of the clerical healers; but it is to be understood that, as far as can be gathered, the medical element is completely submerged by the clerical, and that treatment, and apparently diagnosis also, are entirely in the hands of the latter. In *The American*, a transatlantic paper, appears an account of some of the methods and results of this newest kind of faith-healing. The impression gained from reading a description of how a Massachusetts clergyman treated a dipsomaniac is certainly that hypnotic suggestion pure and simple was employed. The interviewer seems to have been struck by this, and questioned the healer on the subject. The latter admitted that he sometimes employed hypnotism, but denied that he had done so in the particular case. Nevertheless, if the description given is accurate, we think it is quite certain that he did do so.

It is, however, not alone to the neurasthenic and the neurotic that the faith-healers restrict their energies. If that were so, the probability of economic mischief being the upshot of this latest Americanism would be considerably diminished. We are quite prepared to admit that, for many people of the classes just mentioned, it is possible that religious influences may relieve various subjective symptoms of illness; so also may any forms of suggestion or hypnotism, if exercised by those of strong personality over these mentally debilitated people. But we are not by any means prepared to endorse many of the wild statements and hypotheses put forward in all gravity by the reverend protagonists of the Emmanuel idea; in fact, they contain much dangerous and pernicious nonsense. It is held and taught that "physical disease is often only a symptom of deeper distresses growing out of sin and selfishness, and cannot be permanently cured until the deep underlying cause is removed." Now a proposition of this sort is evidently capable neither of proof nor of disproof; but to judge by what is going on in the Emmanuel clinics it is the bedrock foundation of a system of therapeutics which is intended to supplant the usual methods of treating organic as well as functional disease; and if that is so it is a proposition from which we profoundly dissent. How sin and selfishness are to be attacked and removed from the soul, let us say, of a patient delirious with pneumonia is not very evident; but we are solemnly assured that a man is not really cured until his character is changed. We read also of a certain Rev. Mr. Powell, who is treating a lady for a malignant internal growth. It is true we have to accept his diagnosis on this point; and as she has been bedridden for years, the malignancy of the growth cannot be of a very high order. Mr. Powell, it appears, visits this lady and carries out some form of treatment, after which the excruciating pain from which she suffers is relieved for four or five days; during this time she can get up and walk. Now, if Mr. Powell's success in alleviating pain and facilitating locomotion is due to the substitution of "peace, love, and courage for fear, worry, and sin," it is rather difficult to see why relapses should take place at such extremely frequent intervals as five days; for if disease can thus be cured, as is alleged, the malignant internal growth should by now have ceased to exist. We have the clerical gentleman's word for it that a patient "is cured, sometimes instantly, of his sickness or his sin, but usually the treatments must continue for some time." Yet he apparently despairs of ever permanently curing the sin of the lady we have mentioned; for he will not undertake to cure her tumour, but only to relieve her sufferings.

The fact of the matter is that on the subject of their own health few men are qualified to take a calm and unbiassed view. A litigant who would only smile if it were suggested to him that he should replace his barrister and solicitor by a clerk in holy orders to offer up prayers that the judge and jury might be guided aright, will not hesitate to entrust his health to the care of the same person in the capacity of amateur physician. A keen business man, who in daily life makes a point of acting on the best expert advice he can get when he is dealing with a technical matter he does not understand, would not dream of asking a clergyman to pray for a rise in copper or a fall in wheat; at any rate, he would not risk a pennypiece on the experiment. But he will, curiously enough, accept a diagnosis from the same source, and allow his spiritual counsellor to act upon it. We note that he is bidden to have "confidence that ministers have no ulterior or selfish purpose." This thrust at the medical profession in the United States is probably as unjust as the confidence itself would be misplaced. For the sake of the nation at large it may be hoped that Emmanuel clinics will not invade these shores. We have too much belief in the common sense of the main mass of the English clergy and people to believe that this plausible cult of the "priest-physician" could ever obtain a substantial footing; but the medical profession owes it to the public not to let the much advertised doctrines of Mrs. Eddy's latest rivals pass as a workable theory of disease and cure without entering a strong protest to the contrary effect.

